# Low-pH-induced conformational changes in the spike protein promote porcine deltacoronavirus infection by enhancing viral binding efficiency to the porcine aminopeptidase receptor

**DOI:** 10.1128/jvi.01168-26

**Published:** 2026-09-09

**Authors:** Ruiming Yu, Liping Zhang, Peng Zhou, Zhongwang Zhang, Yingjie Bai, Dongsheng Wang, Ya Liu, Xinru Wang, Gang Du, Ligang Yuan, Huichen Guo, Li Pan, Xinsheng Liu

**Affiliations:** 1 State Key Laboratory of Animal Disease Control and Prevention, College of Veterinary Medicine, Lanzhou University, Lanzhou Veterinary Research Institute, Chinese Academy of Agricultural Sciences 111658, Lanzhou, China; 2 Gansu Province Research Center for Basic Disciplines of Pathogen Biology, Lanzhou, China; 3 College of Veterinary Medicine, Gansu Agricultural University 739715, Lanzhou, China; 4 Department of Ophthalmology, The First Hospital of Lanzhou University 117741, Lanzhou, Gansu, China; Loyola University Chicago - Health Sciences Campus, Maywood, Illinois, USA

**Keywords:** gastric acid, PDCoV, low pH, spike, conformation, cryo-electron microscopy

## Abstract

Porcine deltacoronavirus (PDCoV) is a critical enteric pathogen causing severe diarrhea in neonatal piglets, with fecal-oral transmission as its primary route. However, the molecular mechanism underlying PDCoV adaptation to the acidic gastric environment of hosts remains elusive. Here, we demonstrate that low-pH pretreatment (pH 4.0), mimicking the gastric niche of suckling piglets, significantly enhances PDCoV infectivity in porcine intestinal epithelial IPEC-J2 cells, as shown by elevated viral attachment, replication, and progeny titers. Accordingly, neutralization of gastric acid in piglets effectively attenuates PDCoV infection and pathogenicity *in vivo*. Mechanistically, acidic pH induces functionally important conformational rearrangements of the PDCoV spike (S) protein, which markedly increases its binding affinity for the cellular receptor porcine aminopeptidase N (pAPN), with the dissociation constant (KD) decreasing from 20.20 μM at pH 7.2 to 1.69 μM at pH 4.0. We solved the high-resolution cryo-electron microscopy (cryo-EM) structure of the PDCoV S protein under acidic conditions, representing the first deltacoronavirus S structure determined at low pH. Structural comparison with the neutral-pH closed conformation revealed that low pH drives a 14° outward rotation of the S1 C-terminal receptor-binding domain, reducing inter-domain interactions within the trimeric head and generating an intermediate activated conformation between the fully closed and fully open states, while the S2 fusion core remains structurally conserved. This pH-dependent conformational priming is shared by alpha- and deltacoronaviruses with intra-subunit S1 domain packing, which is distinct from the endosomal pH regulation of membrane fusion in betacoronaviruses, and represents an evolutionary adaptation to fecal-oral transmission. Our findings challenge the canonical view of gastric acid solely as a host defense barrier, uncover a preactivation role of the host physiological environment in viral infection, and provide new insights into enteric coronavirus biology, as well as novel targets for prevention and control strategies.

**IMPORTANCE:**

Porcine deltacoronavirus (PDCoV), an emerging porcine enteric coronavirus, poses a serious threat to the swine industry and exhibits potential for cross-species transmission. This study demonstrated that the low-pH environment of the porcine stomach induces specific conformational changes in the PDCoV S protein, thereby maximizing the exposure of its receptor-binding domain, enhancing its binding to the host receptor, and promoting infection of intestinal epithelial cells. Our findings revealed that gastric acid functions not only as a physiological barrier but also as a key "molecular switch" that initiates PDCoV infection and is essential in enabling PDCoV to exploit the host physiological environment and complete its life cycle. These results provide new insights into the precise regulatory mechanisms governing the entry of PDCoV and other porcine enteric viruses, and establish a novel mechanistic basis for vaccine design, drug delivery, and formulation development.

## INTRODUCTION

Coronaviruses (CoVs) are enveloped, single-stranded, positive-sense RNA viruses classified within the order Nidovirales and the family Coronaviridae (1, 2). On the basis of their genomic sequence characteristics, CoVs are classified into four genera: α-, β-, γ-, and δ-CoVs. Bats serve as the natural reservoirs for α- and β-CoVs, whereas birds are the primary natural hosts of γ- and δ-CoVs. Regarding host range, α- and β-CoVs infect primarily mammals, γ-CoVs infect predominantly birds, and δ-CoVs can infect both birds and mammals (1, 3). Human-infecting α- and β-CoVs typically cause respiratory diseases. Of these, the four human CoVs, HCoV-NL63, HCoV-229E, HCoV-OC43, and HKU1, generally induce mild upper respiratory tract infections in immunocompetent people but occasionally cause severe illness in infants and older people. However, since 2000, the emergence of three highly pathogenic CoVs, SARS-CoV, MERS-CoV, and SARS-CoV-2, has substantially threatened global public health and caused extensive social and economic losses (4–6). α-CoVs, such as transmissible gastroenteritis virus (TGEV) and porcine epidemic diarrhea virus (PEDV); β-CoVs, including porcine hemagglutinating encephalomyelitis virus (PHEV) and canine respiratory coronavirus (CRCoV); γ-CoVs, such as infectious bronchitis virus (IBV); and δ-CoVs, such as porcine deltacoronavirus (PDCoV), pose various threats to the healthy development of the livestock industry. Notably, in recent years, newly identified CoVs such as PDCoV have shown potential risks for cross-species transmission to humans (7, 8).

Coronaviruses are the largest RNA viruses currently known. Their virions, consisting of an outer envelope and an inner nucleocapsid, exhibit a crown-like or corona-like appearance under electron microscopy (9). The CoV genome is approximately 27–32 kb long, and it contains 5′ and 3′ untranslated regions, as well as at least six open reading frames (ORFs). These ORFs encode the polymerase protein 1a/pp1b, spike (S) protein, envelope (E) protein, membrane (M) protein, and nucleocapsid (N) protein. Each CoV genome also encodes one or more accessory nonstructural proteins (10–12). Among the structural proteins, S protein is embedded in the viral envelope and forms a homotrimeric structure composed of S1 and S2 subunits (13). The three S1 subunits form the globular head, which is responsible for receptor recognition and binding to host cells, whereas the three S2 subunits constitute the stalk region, which mediates fusion between the viral envelope and the host cell membrane (14, 15). The S1 subunit comprises an N-terminal domain (NTD) and a C-terminal domain (CTD). The NTD exhibits a complex structure and may participate in multiple functions during viral infection, such as mediating immune evasion (15, 16). In contrast, the CTD is generally involved in receptor binding and is therefore also referred to as the receptor binding domain (RBD). The S2 subunit contains several conserved regions, including the fusion peptide (FP), heptad repeat 1 (HR1), heptad repeat 2 (HR2), transmembrane domain, and cytoplasmic tail. The FP inserts into the host cell membrane during early stages of membrane fusion and subsequently initiates the fusion process (17). HR1 and HR2 then interact and form a six-helix bundle structure, thereby bringing the viral envelope and the host cell membrane into proximity, and ultimately driving membrane fusion and enabling viral genome entry into the host cell (18–20). Moreover, the S glycoprotein is highly immunogenic and exerts a crucial role in eliciting neutralizing antibodies. However, the proneness of the gene encoding the S protein to mutation contributes to substantial diversity among CoV species as well as strains of the same species (21, 22).

The CoV S protein, a class I viral membrane fusion protein, mediates membrane fusion after undergoing a series of conformational changes (23). Other class I viral fusion proteins include the hemagglutinin glycoprotein on influenza viruses, the envelope glycoprotein gp160 of human immunodeficiency virus, and the glycoprotein of Ebola virus (24). Class I fusion proteins exist in a metastable prefusion state, whereas the postfusion six-helix bundle exhibits a lower-energy conformation. Accordingly, the transition from prefusion to postfusion state is energetically favorable. This conformational change can be triggered by receptor binding, pH alterations, or both, each of which plays an important role in the fusion process (25, 26). According to structural analyses, S trimers of β-CoVs (including SARS-CoV, MERS-CoV, and SARS-CoV-2, as well as the γ-CoV IBV) adopt both "up" and "down" conformations (25, 27, 28). In contrast, S trimers from α-CoVs such as HCoV-NL63 and PEDV, and from PDCoV, have been observed exclusively in the "down" conformation (29–31). For the α-CoV HCoV-229E, two distinct trimeric conformations have been identified: a compact state and a relaxed state. In the relaxed conformation, the steric hindrance that restricts the transition of the CTD from the "down" to the "up" state is diminished, thereby facilitating receptor binding (32).

The reported crystal structure of the PDCoV RBD in complex with aminopeptidase N (APN) has revealed that residues within the β5–β6 loop (N397-Y398-L399-L400-R401) specifically mediate interaction with APN (33). However, how the spike protein of δ-CoV undergoes the conformational changes that expose its RBD for receptor binding remains unclear. A thorough understanding of the mechanisms through which the S trimer, within the quaternary assembly of S1 subunits, interacts with host receptors and undergoes accompanying conformational changes will be essential for guiding the rational design of antiviral interventions against CoVs. Low-pH environments have been reported to promote δ-CoV entry in a specific manner (34). Herein, low pH was found to trigger conformational changes in the PDCoV S protein, thereby significantly enhancing its binding to APN and promoting viral infectivity. Our findings revealed the dynamic conformational rearrangements of the δ-CoV S trimer under low-pH conditions, thus providing mechanistic insights into receptor binding and the transition from the prefusion to the postfusion state. Our results might also contribute to a more integrated understanding of the dynamic interplay among the host, pathogen, and environmental factors.

## RESULTS

### Low-pH pretreatment significantly enhances the infectivity of PDCoV in intestinal epithelial cells

**Fig 1:**
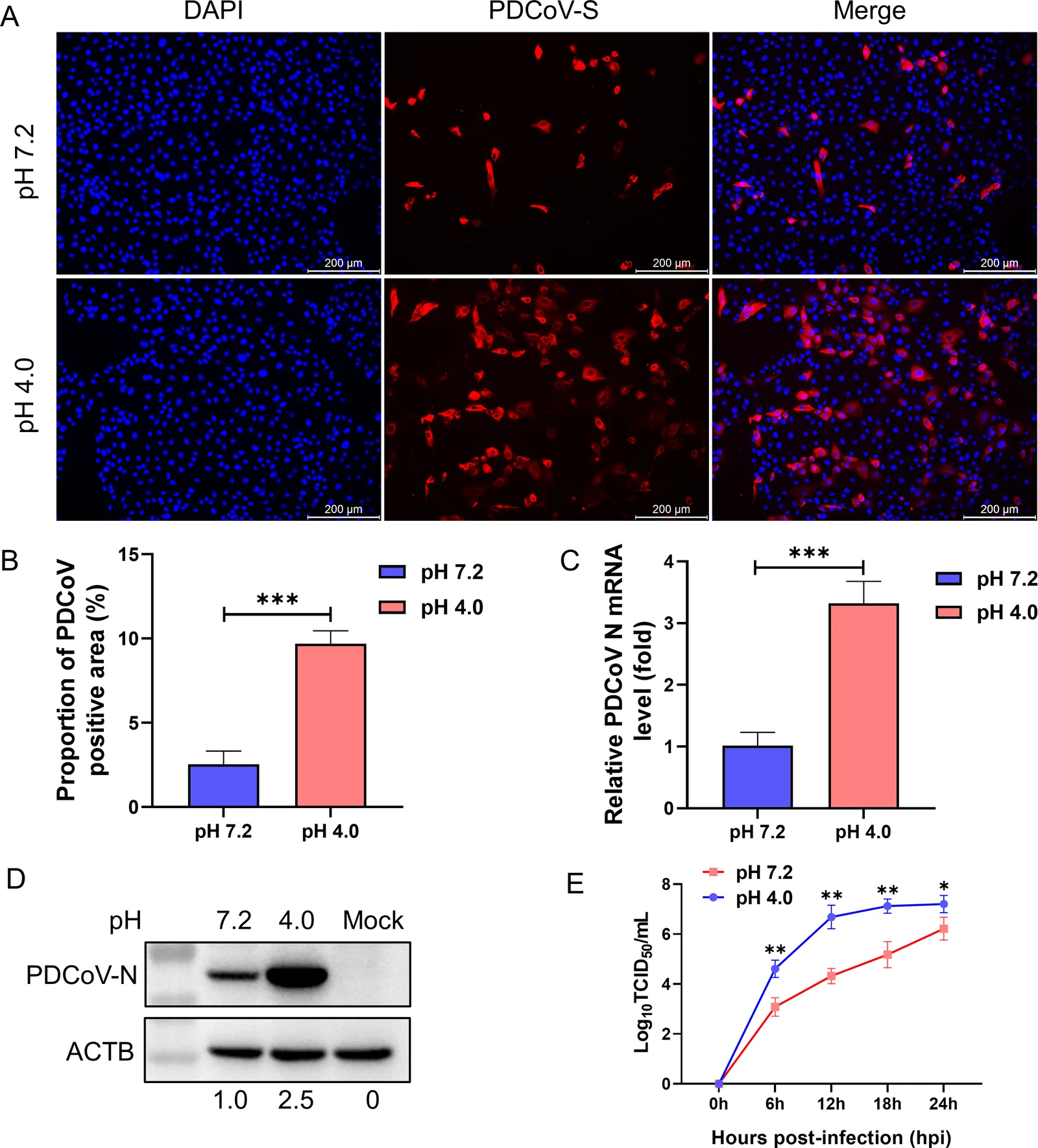
Low-pH (pH 4.0) promotes PDCoV infection in host cells. (**A**) The proliferation of PDCoV in IPEC-J2 cells was detected with IFA. IPEC-J2 cells were infected with PDCoV at MOI = 0.01 after treatment with PBS at pH = 4.0 or pH = 7.2 and neutralization. After 18 h, the cells were fixed with 4% paraformaldehyde, and IFA was performed with rabbit anti-S polyclonal antibody as the primary antibody and Alexa-Fluor-594-conjugated goat anti-rabbit IgG as the secondary antibody. (**B**) The proportion of positive cell area in IFA was calculated in ImageJ. (**C**) The expression of PDCoV N RNA in IPEC-J2 cells was detected with RT-qPCR. IPEC-J2 cells were infected with PDCoV at MOI = 0.01 after treatment with PBS at pH = 4.0 or pH = 7.2 and neutralization. After 18 h, the cell samples were collected, and RNA was extracted. The RNA expression of the PDCoV N gene in IPEC-J2 cells was detected with RT-qPCR. (**D**) The expression of PDCoV N protein in IPEC-J2 cells was detected with western blotting. IPEC-J2 cells were infected with PDCoV at MOI = 0.01 after treatment with PBS at pH = 4.0 or pH = 7.2 and neutralization. Cell samples were collected 18 h later. Western blot analysis was performed with rabbit anti-N polyclonal antibody as the primary antibody and HRP-conjugated goat anti-rabbit IgG as the secondary antibody. (**E**) Growth kinetics of PDCoV in IPEC-J2 cells. Viral titers in culture supernatants were measured by TCID_50_ assay at the indicated time points. Data in B, C, and E are shown as mean ± standard deviation from three independent biological replicates. Statistical significance was determined by unpaired two-tailed *t*-tests. *, *P* < 0.05; **, *P* < 0.01; ***, *P* < 0.001.

To investigate the regulatory effect of low-pH pretreatment on PDCoV infectivity, PDCoV was pretreated with PBS at pH 7.2 (neutral control group) or pH 4.0, followed by neutralization, and then used to inoculate IPEC-J2 cells at the same infectious dose. At 18 h post-infection, viral infection levels were assessed using multiple assays: indirect immunofluorescence imaging and quantitative analysis showed that compared with the pH 7.2 control group, the pH 4.0 pretreatment group exhibited a wider distribution of viral fluorescence signals and a higher proportion of positive cells, and the percentage of virus-positive signal area was significantly elevated with a statistically significant difference (*P* < 0.001) (Fig. 1A and B); RT-qPCR quantification revealed that the relative RNA expression level of the PDCoV N gene in the pH 4.0 pretreatment group was markedly higher than that in the pH 7.2 control group, reaching an extremely significant difference (*P* < 0.001) (Fig. 1C), confirming that low-pH treatment substantially enhances the replication level of PDCoV in intestinal epithelial cells; Western blot analysis with β-actin (ACTB) as the loading control showed that the band intensity of PDCoV N protein in the pH 4.0 group was obviously stronger than that in the pH 7.2 group (Fig. 1D), with uniform and stable loading control bands, further verifying the infection-enhancing effect of low-pH pretreatment at the protein level; we subsequently determined the 50% tissue culture infectious dose (TCID_50_) of PDCoV in IPEC-J2 cells at multiple time points post-infection for both groups, and the results showed that TCID_50_ titers in the pH 4.0 pretreatment group were significantly higher than those in the pH 7.2 control group at 6 h, 12 h, 18 h, and 24 h post-infection (*P* < 0.05) (Fig. 1E). Taken together, these findings demonstrate that low-pH pretreatment at pH 4.0 significantly enhances the infectivity of PDCoV in porcine intestinal epithelial cells.

### Neutralization of gastric acidity effectively inhibits PDCoV infection in suckling piglets

**Fig 2:**
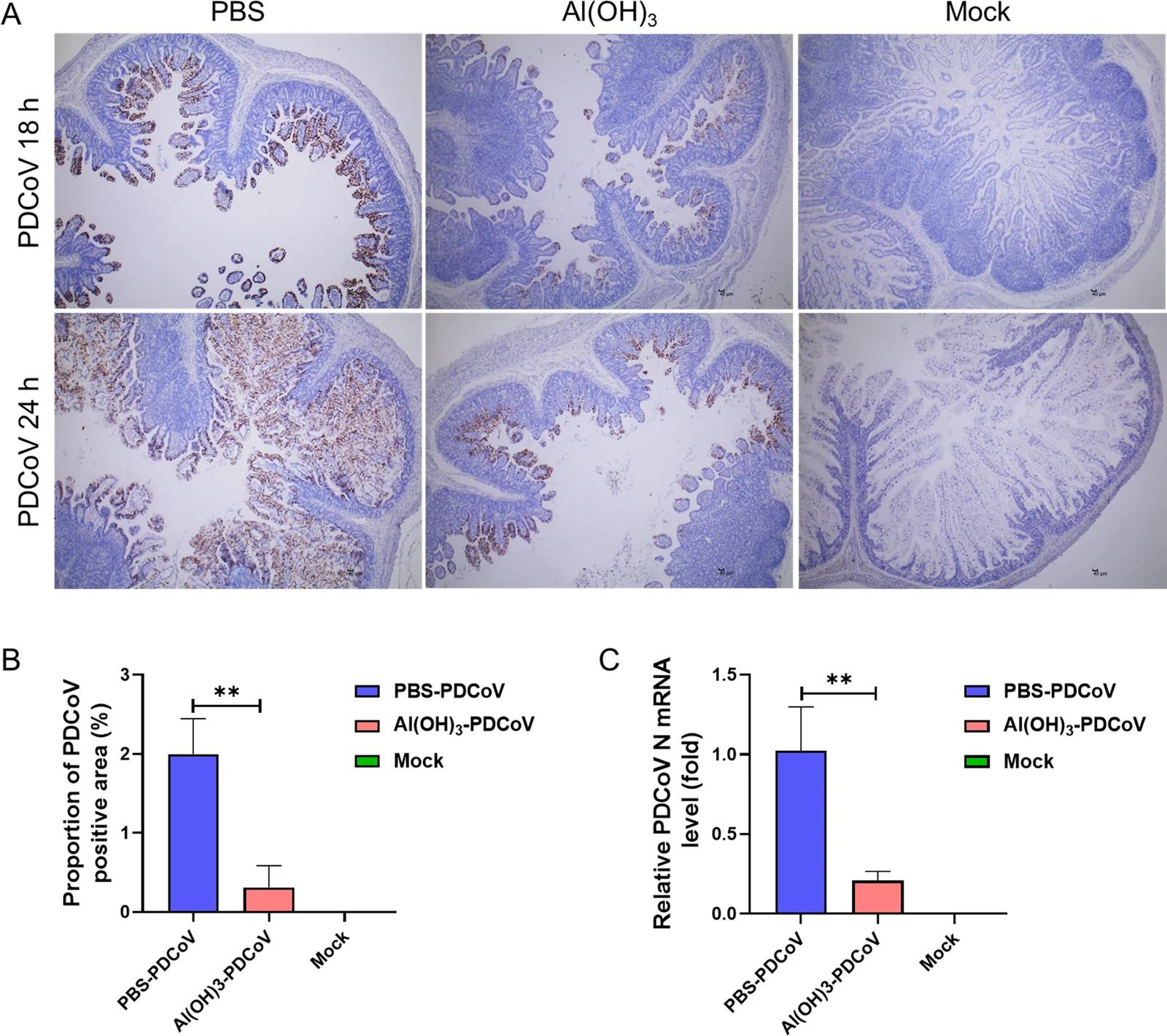
Neutralizing gastric acid effectively inhibits PDCoV infection in suckling piglets. (**A**) The intestinal tissue of the euthanized piglets was examined by immunohistochemistry 18 h and 24 h after challenge. The PDCoV antigen signal was visible as brown coloration. (**B**) The proportion of PDCoV-positive signal area in each group in immunohistochemistry was analyzed in ImageJ. (**C**) The expression of PDCoV N RNA in intestinal tissue was detected with RT-qPCR. Data are shown as mean ± standard deviation from three independent biological replicates. Statistical significance was determined by unpaired two-tailed *t*-tests. *, *P* < 0.05; **, *P* < 0.01; ***, *P* < 0.001.

22 five-day-old suckling piglets were randomized into three groups: a treated group (*n* = 8), an untreated group (*n* = 8), and a mock control group (*n* = 6). Piglets in the treated group were orally administered Al(OH)₃ gel three times (3 mL per dose per piglet), whereas those in the untreated group received PBS, and those in the mock control group received no treatment. After three administrations of Al(OH)₃ gel, the gastric pH of treated piglets increased to approximately 6.9, a value exceeding the pH of approximately 4.5 in PBS-treated piglets. At 18 h and 24 h post-challenge, immunohistochemistry revealed markedly lower PDCoV antigen levels in intestinal tissues in the Al(OH)₃ group than the PBS group. No viral antigen was detected in the mock control group because those animals were not challenged (Fig. 2A and B). Moreover, reverse transcription quantitative PCR (RT-qPCR) analysis indicated significantly lower PDCoV RNA levels in the intestinal tissues of Al(OH)₃-treated piglets than PBS-treated piglets, whereas no viral RNA was detected in the mock control group (Fig. 2C). These findings indicated that neutralization of gastric acidity, resulting in elevated gastric pH, effectively suppressed PDCoV infection in suckling piglets. Therefore, a mildly acidic gastric environment might facilitate PDCoV infection *in vivo*.

### Low-pH promotes viral cell attachment by enhancing the binding efficiency of spike protein to pAPN

**Fig 3:**
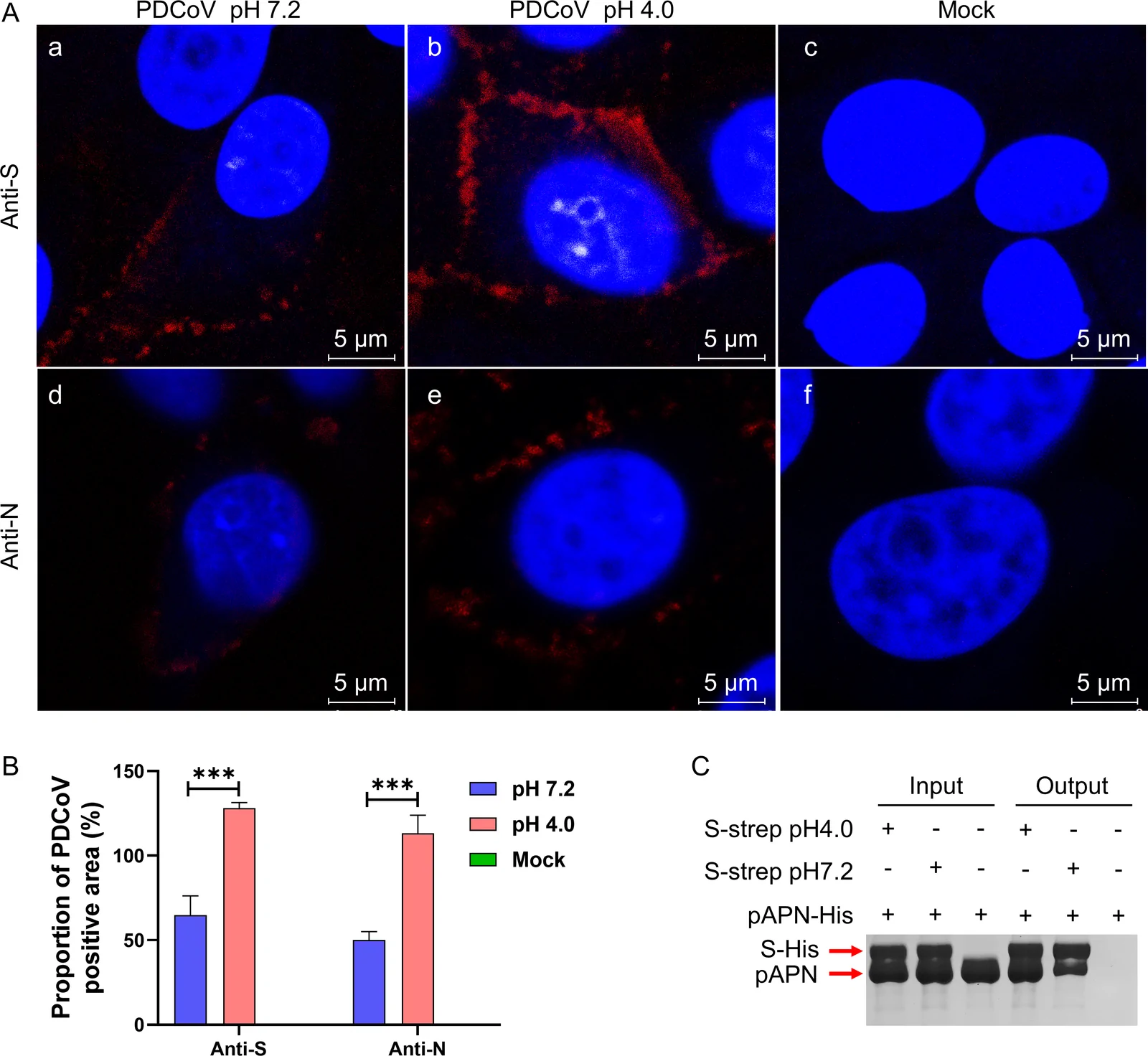
Low-pH promotes PDCoV attachment to IPEC-J2 cells and enhances the binding of S protein to the pAPN receptor. PDCoV virions were pretreated with buffer at pH 7.2 or pH 4.0, neutralized, and then incubated with IPEC-J2 cells at 4°C for 1.5 h to allow viral attachment to the cell surface while blocking endocytosis. (**A**) Confocal fluorescence images of membrane-bound PDCoV particles. Virions were detected with rabbit anti-S polyclonal antibody (upper panels) or rabbit anti-N polyclonal antibody (lower panels) in red, and cell nuclei were counterstained with DAPI in blue. Mock-infected cells served as a negative control. Scale bars, 5 μm. (**B**) Quantitative analysis of the percentage of PDCoV-positive area on the cell surface. Data represent means ± standard deviations from three independent biological replicates. ***, *P* < 0.001 as determined by unpaired two-tailed *t*-tests. (**C**) *In vitro* pull-down assay for the S-pAPN interaction. Strep-tagged PDCoV S proteins pretreated at pH 4.0 or pH 7.2 were immobilized on streptactin agarose resin and incubated with His-tagged pAPN protein. Input proteins and eluted protein complexes were separated by SDS-PAGE for analysis.

**Fig 4:**
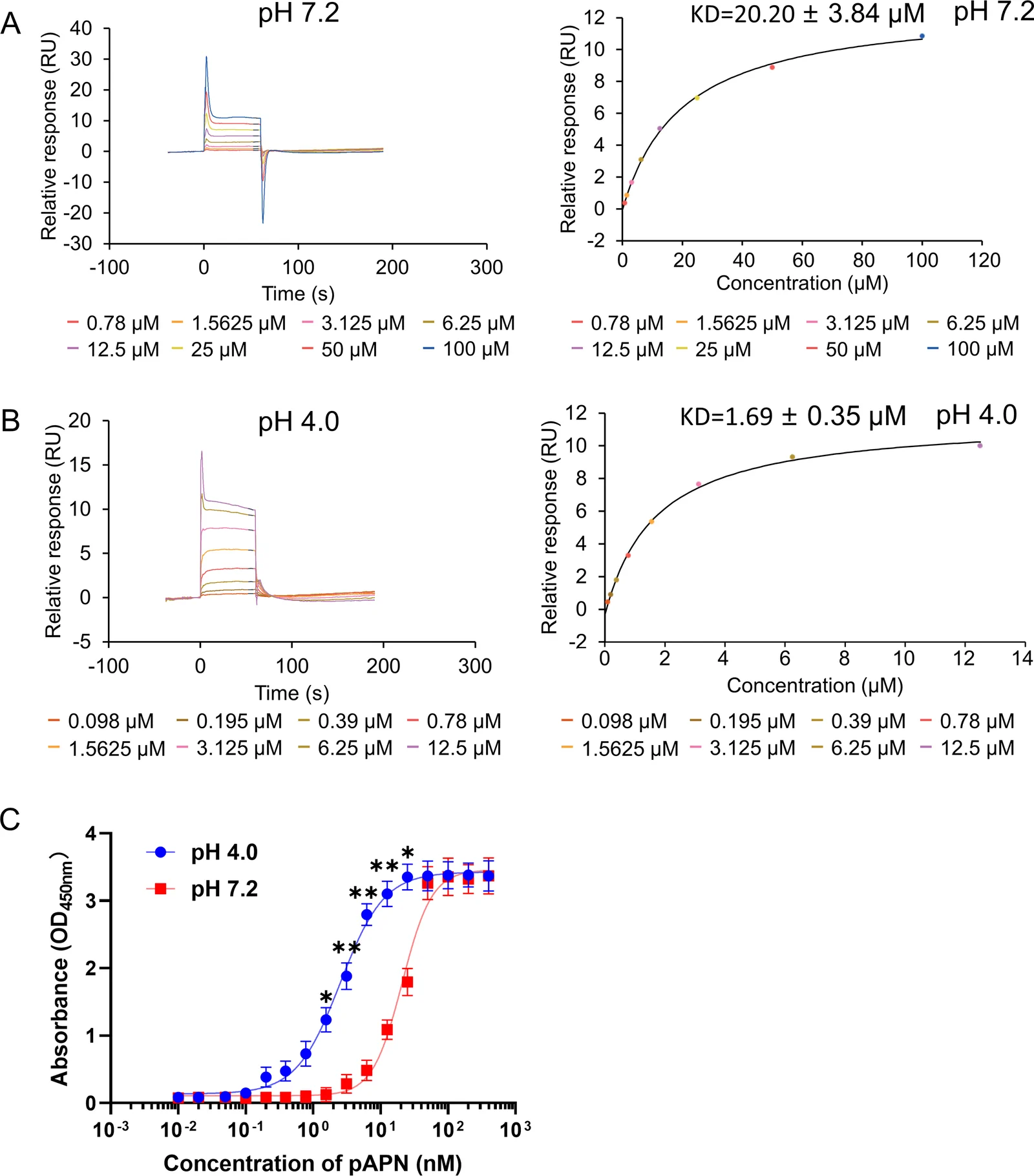
Low-pH treatment significantly enhances the binding affinity of PDCoV S protein to the pAPN receptor. (**A and B**) SPR analysis of the binding kinetics between pAPN and PDCoV S protein pretreated at pH 7.2 (**A**) or pH 4.0 (**B**). Left panels: representative sensorgrams of S protein binding to immobilized pAPN at serial concentrations. Right panels: steady-state affinity fitting curves for dissociation constant (KD) calculation. The KD value was determined to be 20.20 ± 3.84 µM at pH 7.2 and 1.69 ± 0.35 µM at pH 4.0. (**C**) ELISA validation of S-pAPN binding activity. Gradient concentrations of pAPN were incubated with immobilized S proteins pretreated at different pH values, and absorbance at 450 nm was measured to reflect binding capacity. Data are shown as mean ± standard deviation from three independent replicates. Statistical significance was determined by unpaired two-tailed *t*-tests. *, *P* < 0.05; **, *P* < 0.01; ***, *P* < 0.001.

PDCoV was separately treated with PBS at pH 4.0 or pH 7.2, then neutralized and used to infect IPEC-J2 cells. The cells were incubated at 4°C for 1.5 h to allow viral attachment while preventing internalization (35). Subsequently, PDCoV particles on the plasma membrane were imaged by high-resolution microscopy, with cell nuclei counterstained with DAPI and viral particles labeled with either rabbit anti-S polyclonal antibody or rabbit anti-N polyclonal antibody to eliminate detection bias caused by conformational changes that could alter recognition by a single antibody. The results showed that compared with the pH 7.2 control group, the number of viral particles on the cell surface was markedly increased in the pH 4.0 acidic treatment group. Quantitative analysis revealed that the percentage of virus-positive signal area was extremely significantly elevated in the pH 4.0 group (*P* < 0.001), and consistent trends were observed for both detection antibodies (Fig. 3A and B), indicating that low-pH treatment effectively promotes the attachment of PDCoV to host cells. *In vitro* pull-down assays demonstrated that with equivalent protein input, S protein pretreated at pH 4.0 pulled down substantially more pAPN protein, directly confirming that acidic conditions strengthen the interaction between S protein and pAPN (Fig. 3C). We further quantified the binding affinity between the two proteins via surface plasmon resonance (SPR). Kinetic analysis showed that the dissociation constant (KD) of S protein to pAPN was 20.20 ± 3.84 µM at pH 7.2, while pH 4.0 treatment significantly enhanced the binding affinity with a notably reduced KD value (Fig. 4A and B). In line with this, indirect ELISA binding assays showed that across all tested gradient concentrations of pAPN, the OD450 nm values of the pH 4.0 group were consistently higher than those of the pH 7.2 group, exhibiting dose-dependent enhancement of binding activity (Fig. 4C). Collectively, these multi-dimensional experimental results consistently demonstrate that low pH significantly increases the binding efficiency of PDCoV S protein to the pAPN receptor, an effect that may be mediated by low-pH-induced conformational rearrangements of the S protein trimer.

### Cryo-EM structure of low-pH treated prefusion PDCoV spike

Building on the biochemical finding that low pH significantly enhances the binding affinity of the PDCoV S protein to its receptor pAPN, we next sought to resolve the underlying structural basis of this effect. The ectodomain trimer of PDCoV S protein was expressed and purified using the ExpiCHO eukaryotic expression system, then treated with pH 4.0 buffer for structural analysis. Preliminary negative-stain transmission electron microscopy (TEM) showed that S protein remained intact under acidic conditions, with no aggregation, degradation, or distortion. The protein formed a uniform population of cone-shaped monodisperse particles, indicating sufficient stability for high-resolution structural determination (Fig. S1).

We collected single-particle cryo-electron microscopy (cryo-EM) data of the low-pH-treated S protein. After screening 9,408 micrographs, a total of 993,371 valid particles were selected for 3D reconstruction and refinement (Fig. S2). We finally resolved the prefusion conformation of PDCoV S protein under pH 4.0 (designated S4.0) at an overall resolution of 2.83 Å. The structure covers amino acid residues D20 to N1077, and the local resolution of core functional domains meets the requirements for atomic model building (Fig. S3).

The overall structure of S4.0 adopts a typical homotrimeric clover-shaped conformation, with three S1 subunits capped on top of the S2 stalk. The S1 subunit is dominated by β-sheets and consists of the N-terminal domain (NTD, residues D54–V277), C-terminal receptor-binding domain (S1-CTD/RBD, residues L302–A422), subdomain 1 (SD1, residues C433–P483), and subdomain 2 (SD2, residues P487–T553). The α-helix-rich S2 subunit contains core membrane fusion elements including the fusion peptide (FP, residues R669–D695), heptad repeat 1 (HR1, residues F753–E854), and central helix (CH, residues E855–K903) (Fig. S4).

### Low-pH triggers an open conformation of the spike trimer

**Fig 5:**
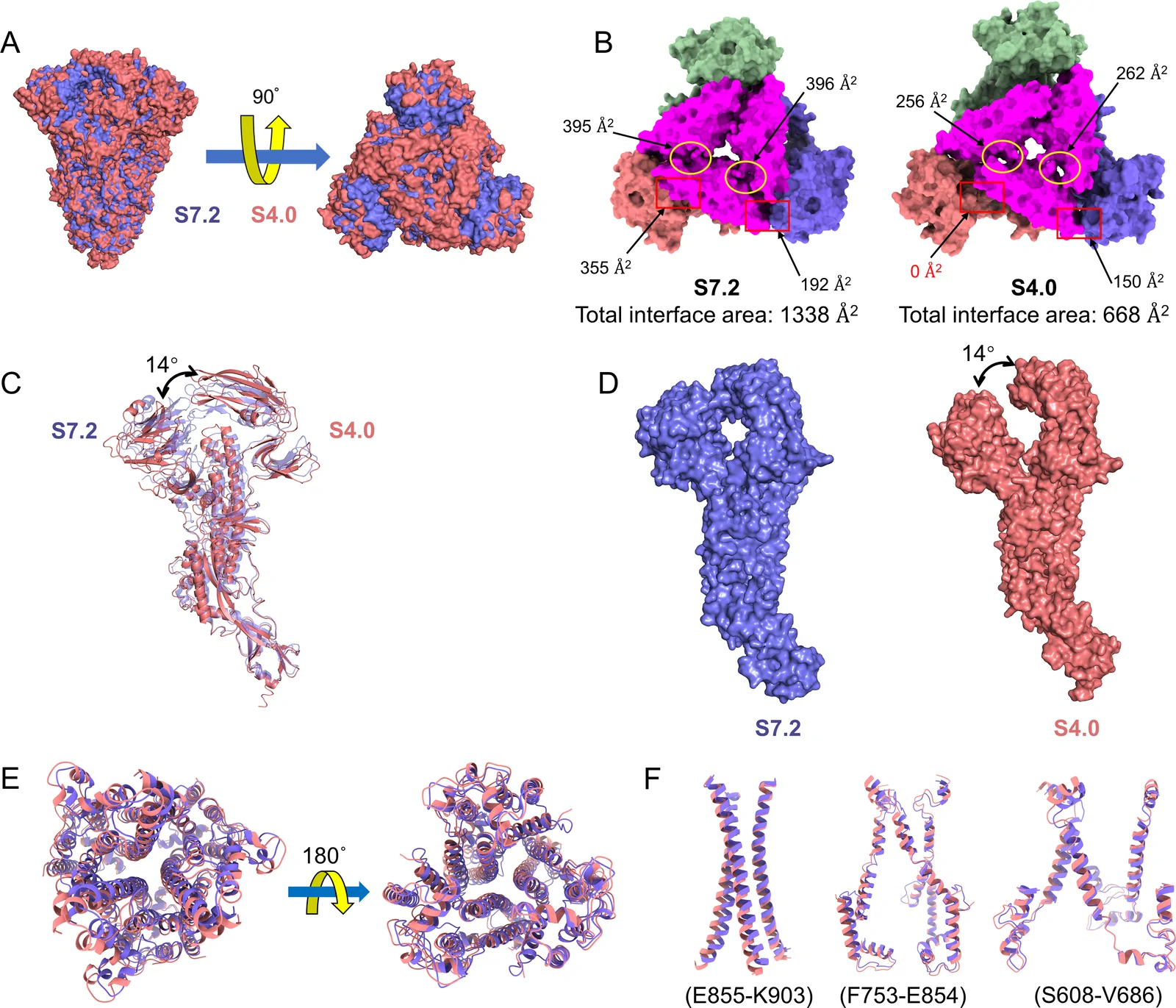
The PDCoV S trimer has an open structure at low-pH (pH 4.0). (**A**) The structure of PDCoV S protein S4.0 at low pH (pH 4.0), compared with the structure of S7.2 under physiological conditions. S4.0 is in salmon red, and S7.2 is in sky blue. (**B**) The interface areas between the S1-CTD (chain A) and the surrounding S1-NTDs and S1-CTDs from adjacent monomers are shown. The interface areas were analyzed with PDBePISA. The S1-NTDs of the A, B, and C chains are in salmon red, light green, and sky blue, respectively. The S1-CTDs are in magenta. (**C, D**) The angle between S1-CTD and S1-NTD of the S4.0 monomer increased by 14°. Structures in E are shown in cartoon representation, and structures in F are shown in surface representation. The S7.2 monomer is in sky blue, and the S4.0 monomer is in salmon red. (**E**) Overall structural comparison of the S2 subunit from S4.0 and S7.2. S7.2 is in sky blue, and S4.0 is in salmon red. (**F**) Structural comparison of structural elements in the S2 subunit.

To characterize the conformational changes induced by acidic conditions, we performed a global structural alignment of S4.0 with S7.2, a homology model of PDCoV S protein at neutral pH generated using the previously reported physiological structure (PDB: 6B7N) as the template. The results revealed clear differences in the overall backbone between the two structures: the trimer head of S4.0 is more extended and adopts a more open conformation. Conformational changes were predominantly localized to the S1 subunit, while the core fusion elements of the S2 subunit showed no significant alteration (Fig. 5A).

Further quantitative analysis of inter-domain interactions showed that the interface area between S1-CTD of one protomer and its adjacent S1-NTD, as well as S1-CTD from the neighboring protomer, was markedly smaller in S4.0 than in S7.2, indicating weakened inter-domain interactions (Fig. 5B). Measurement of the angle between S1-CTD and S1-NTD within a single protomer showed that S1-CTD underwent a 14° outward rotation under low-pH conditions. The separation of the two originally tightly bound domains directly resulted in conformational loosening of the trimer head (Fig. 5C and D). In contrast, core structures of the S2 subunit (FP, HR1, and CH) were highly conserved between the two pH conditions, with no notable conformational shift. This finding suggests that low pH only induces pre-activating conformational changes related to receptor binding and does not trigger irreversible conformational transitions associated with membrane fusion (Fig. 5E and F).

### Open S1-CTD relieves steric hindrance for pAPN binding

**Fig 6:**
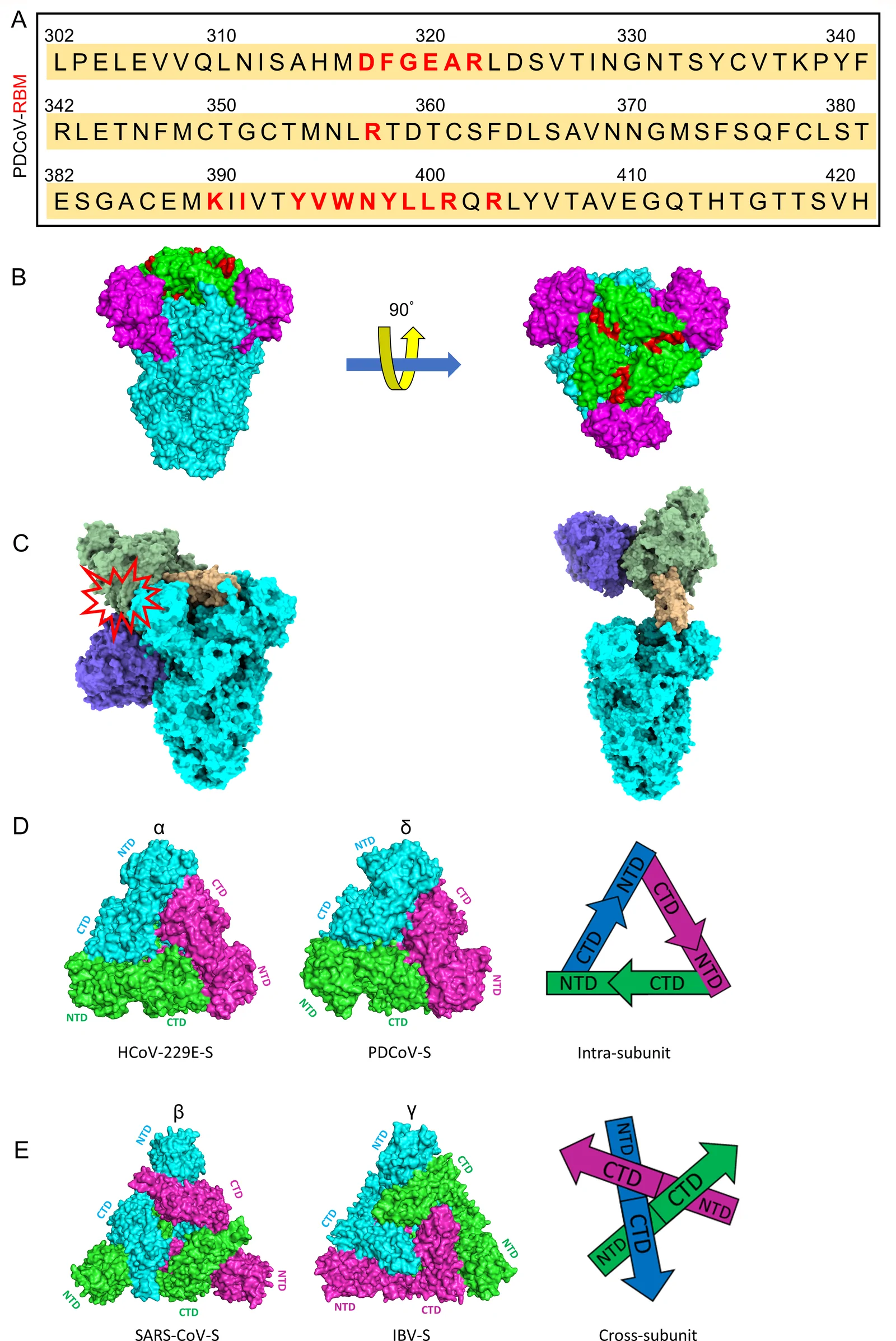
PDCoV S1-CTD/RBD must be converted to the “up” conformation to bind the receptor pAPN. (**A**) Amino acid sequence of PDCoV S-CTD/RBD. The red-tagged amino acid residue is PDCoV-RBM. (**B**) PDCoV-RBM is embedded in the S1 subunit. PDCoV-RBM is in red. (**C**) Left panels: superposition of the complex structure of S-CTD/RBD and pAPN (PDB ID: 7VPP) with the S trimer structure (PDB ID: 6B7N). When S-CTD/RBD is in the “down” conformation, substantial steric hindrance affects pAPN binding. Right panels: after conversion of the PDCoV S-CTD/RBD to the “up” conformation, the steric hindrance disappears, thus enabling pAPN binding. The PDCoV S trimer is in cyan, the pAPN dimer is in light green and sky blue, and the S-CTD/RBD bound to pAPN is in wheat. (**D**) The close interaction between S1-CTD and S1-NTD in the same chain of α- and δ-CoVs indicates an intra-subunit accumulation model. (**E**) The interaction between S1-CTD and S1-NTD in the adjacent chains of β- and γ-CoVs indicates an inter-subunit stacking pattern. α-CoV HCoV-229E S trimer, PDB ID: 7CYC; δ-CoV PDCoV S trimer, PDB ID: 6B7N; β-CoV SARS-CoV S trimer, PDB ID: 6ACC; and γ-CoV IBV S trimer, PDB ID: 6CV0. The A, B, and C chains of all S trimers are in green, cyan, and magenta, respectively.

To elucidate the functional significance of S1 conformational opening, we analyzed its regulatory effect on receptor binding based on the reported crystal structure of the PDCoV RBD-pAPN complex. Spatial mapping showed that the majority of the PDCoV receptor-binding motif (RBM) is buried inside the S1 subunit, with only a small region exposed on the trimer surface (Fig. 6A and B). Molecular docking simulations demonstrated that when S1-CTD is in the closed (down) conformation under neutral conditions, severe steric hindrance prevents pAPN from accessing the RBM. Efficient receptor binding only occurs when S1-CTD switches to the fully open (up) conformation, which eliminates the steric clash (Fig. 6C).

The 14° outward rotation of S1-CTD in the S4.0 structure, while not forming a full up conformation, greatly reduces the spatial constraint imposed by S1-NTD on S1-CTD and lowers the energy barrier for its transition to the up state. This conformational change facilitates RBM exposure and subsequent pAPN binding, which is fully consistent with our biochemical observation that low pH enhances S protein-receptor binding efficiency. Further analysis of the quaternary assembly patterns of coronavirus S proteins showed that α- and δ-coronaviruses adopt an intra-subunit packing mode: S1-CTD and S1-NTD from the same polypeptide chain interact tightly, locking the RBD inside the trimer and preventing spontaneous conversion to the up conformation (Fig. 6D). In contrast, β- and γ-coronaviruses use an inter-subunit packing mode, in which RBDs are more prone to conformational flipping (Fig. 6E). This divergence indicates that low-pH-induced S1 conformational loosening is a unique regulatory mechanism for receptor binding in α/δ-coronaviruses with the intra-subunit packing pattern.

## DISCUSSION

In this study, we systematically demonstrate that low-pH conditions mimicking the gastric environment of suckling piglets significantly enhance the infectivity of PDCoV, whereas neutralization of piglet gastric acid effectively inhibits the *in vivo* infection and pathogenicity of the virus. Mechanistically, the acidic environment induces functionally relevant conformational rearrangements of the PDCoV S protein, which promotes viral attachment by increasing the binding efficiency of the S protein to its receptor pAPN and ultimately boosts viral infectivity. This finding challenges the traditional view that gastric acid serves solely as a host defense barrier and reveals that the host physiological environment can act as a programmatic activation signal for viral infection, providing a new perspective for understanding the infection cycle of enteric coronaviruses.

This study presents the first high-resolution structure of a deltacoronavirus S protein under acidic conditions, filling an important gap in the field. Compared with the previously reported closed conformation of the PDCoV S protein at neutral pH (31), low pH induces a 14° outward rotation of the S1-CTD/RBD, resulting in a more open trimeric conformation that represents an intermediate activated state between the closed state and the fully open state. In terms of regulatory mode, the pH-dependent conformational regulation of PDCoV differs markedly from that of betacoronaviruses: for respiratory betacoronaviruses such as SARS-CoV-2, pH-dependent regulation occurs primarily at the endosomal stage after endocytosis, with a core role in mediating membrane fusion (36–38); in contrast, as an enteric coronavirus, PDCoV undergoes pH-dependent regulation in the extracellular gastric environment as a pre-activation step prior to cell entry, representing an adaptive evolution of the virus to the fecal-oral transmission route. Furthermore, this conformational loosening pattern is highly similar to the regulatory features of HCoV-229E, an alphacoronavirus that also uses APN as its receptor (32), suggesting that APN-dependent coronaviruses may share a conserved low-pH preactivation mechanism. In light of the two quaternary packing modes of coronavirus S proteins, alpha- and deltacoronaviruses adopt an intra-subunit packing mode, in which S1-CTD is tightly constrained by the *cis* S1-NTD, making it difficult to form the fully “up” conformation typical of betacoronaviruses. Accordingly, relieving steric hindrance via conformational loosening is the primary mechanism by which these viruses regulate receptor binding.

The “gastric acid preactivation” mechanism identified in this study carries clear physiological significance and evolutionary implications. PDCoV infects neonatal piglets via the fecal-oral route, and the gastric juice of suckling piglets maintains a weakly acidic pH ranging from 4.0 to 6.0. This pH range is sufficient to induce conformational loosening of the S protein without causing its denaturation and inactivation, thereby priming the virus for rapid receptor binding and initiation of infection once it reaches the intestinal tract. This strategy of exploiting the host physiological environment to activate its own infectivity is a result of long-term co-evolution between the virus and its host and also explains the high infectivity of PDCoV in neonatal piglets. In addition, this mechanism may be conserved among multiple porcine enteric coronaviruses such as PEDV and TGEV, providing a new entry point for systematically understanding the regulatory rules of enteric coronavirus infection.

Our findings also provide a fundamental rationale for the development of PDCoV prevention and control technologies. In terms of vaccine development, the design of oral vaccines should fully account for the effect of gastric acid on S protein conformation. Strategies such as antigen encapsulation and conformational stabilization modifications can be applied to prevent premature antigen activation by gastric acid and preserve its immunogenicity. For injectable vaccines such as inactivated vaccines, the intermediate conformation under acidic conditions can be referenced to design immunogens and improve the induction efficiency of neutralizing antibodies. In terms of antiviral drug development, small-molecule inhibitors that stabilize the S protein conformation and block low-pH-induced conformational loosening may serve as novel targets for anti-PDCoV drug development. Furthermore, this mechanism also provides a reference for research on the prevention and control of other fecal-orally transmitted enveloped viruses.

This study has certain limitations. Currently, we have only resolved the structure of the PDCoV S protein at a single pH condition (pH 4.0), and have not systematically investigated the gradient regulatory effects of different pH gradients on S protein conformation and viral infectivity, which cannot fully cover the gastric environment under different physiological states. Future studies will further resolve the S protein structures across multiple pH gradients, and combine molecular dynamics simulations to reveal the dynamic conformational regulatory process of the S protein in response to pH changes. Meanwhile, we will verify the conservation of this low-pH preactivation mechanism in other enteric coronaviruses, to provide theoretical support for the development of broad-spectrum anti-enteric coronavirus strategies.

## MATERIALS AND METHODS

### Cells and virus

The IPEC-J2 cell line was preserved at the State Key Laboratory of Veterinary Etiological Biology, Lanzhou Veterinary Research Institute, Chinese Academy of Agricultural Sciences. Cells were cultured in Dulbecco’s modified Eagle’s medium supplemented with 10% fetal bovine serum at 37°C in a humidified incubator with 5% CO₂. ExpiCHO-S cells (Thermo Fisher Scientific) were cultured in ExpiCHO expression medium (Thermo Fisher Scientific) at 37°C under 8% CO₂ in a shaking incubator at 125 rpm. The PDCoV strain CH/XJYN/2016 (PDCoV CH/XJYN/2016) was isolated and preserved in the laboratory (39). The virus was propagated in IPEC-J2 cells cultured in minimum essential medium supplemented with 20 μg/mL trypsin.

### Plasmid construction

The N-terminal signal peptide and the C-terminal transmembrane region of the S protein were removed, whereas amino acid residues 20-1077 were retained. A heterologous signal peptide derived from secretoglobin family 1D member 1 (sequence MRLSVCLLLLTLALCCYRANA) was introduced at the N-terminus. At the C-terminus, a T4 fibritin trimerization motif, a human rhinovirus 3C (HRV 3C) protease cleavage site, an 8× His tag, and a Strep-tag II were sequentially appended. The N-terminal transmembrane region of pAPN (NP_999442.1) was removed, and a 6× His tag was added at the C-terminus. Subsequently, the PDCoV S and pAPN genes were cloned into the pcDNA3.1(+) expression vector.

### Protein expression and purification

As previously described (40), recombinant plasmids were transfected into ExpiCHO-S cells according to the manufacturer’s instructions for the ExpiCHO expression system. The cells were cultured at 32°C under 5% CO₂ in a shaking incubator at 125 rpm for 10–12 days. The culture supernatant was harvested by centrifugation at 4,000–5,000 × *g* for 30 min at 4°C in a refrigerated centrifuge, then filtered through a 0.22 μm membrane. The clarified supernatant was subjected to affinity purification with Ni-NTA agarose (Qiagen, Germany). Further purification of S protein was performed through size-exclusion chromatography with a Superose 6 10/300 GL column. The purified protein was stored in 1× PBS supplemented with 20% glycerol, divided into aliquots, and stored at −80°C until use.

### Indirect immunofluorescence assays

IPEC-J2 cells were seeded in six-well plates and cultured overnight. The cells were then infected with PDCoV at a multiplicity of infection (MOI) of 0.01. At 18 h post-infection, cells were washed three times with PBS and fixed with 4% paraformaldehyde for 1 h. After removal of paraformaldehyde, the cells were permeabilized with 0.25% Triton X-100 at room temperature for 10 min, then blocked with 5% bovine serum albumin (BSA) for 1 h. The cells were then incubated with rabbit anti-PDCoV S protein polyclonal antibody or anti-PDCoV N protein polyclonal antibody as the primary antibody, then with Alexa Fluor 594-conjugated goat anti-rabbit IgG as the secondary antibody. Nuclei were stained with DAPI for 5 min in the dark. After three washes with PBS, cells were observed under a fluorescence microscope.

### Western blot analysis

Protein samples were separated with polyacrylamide gel electrophoresis (PAGE) and subsequently transferred onto nitrocellulose membranes (Pall, USA). The membranes were incubated with rabbit anti-PDCoV-N polyclonal antibody and β-actin (ACTB) mouse monoclonal antibody (Origene, China) as the primary antibodies, then with horseradish peroxidase (HRP)-conjugated goat anti-rabbit IgG or goat anti-mouse IgG (ZSGB-Bio, China) as the secondary antibodies. Protein bands were visualized with an enhanced chemiluminescence substrate (Thermo Scientific, USA).

### RT-qPCR

Total RNA was extracted with an Eastep Super Total RNA Extraction Kit (Promega, LS1040). RT-qPCR was performed with a One-Step TB Green PrimeScript RT-PCR Kit (TaKaRa, RR086A). All reactions were conducted in triplicate, and the data were normalized to the expression level of β-actin (ACTB) in each sample. Melting curve analysis confirmed the formation of a single specific product in all cases. Relative changes in gene expression were calculated with the 2^−ΔΔCt^ method.

### TCID_50_

The virus suspension harvested via freeze-thaw was thawed at room temperature and centrifuged at 3,500 rpm for 2 min, after which the supernatant was collected for titration. Serial 10-fold dilutions of the virus (10⁻¹, 10⁻², ...; the dilution range was determined based on the estimated virus titer) were prepared using MEM medium supplemented with trypsin at a final concentration of 20 μg/mL. Cells were seeded into 96-well plates with a multichannel pipette to reach approximately 80% confluence, then washed twice with PBS followed by one wash with serum-free MEM. For each virus dilution, 100 μL was added to the wells, with eight replicate wells set per dilution. An additional 100 μL of MEM was supplemented to each well to achieve a final volume of 200 μL. Wells containing 200 μL of MEM alone were set as the blank control. The plates were incubated at 37°C. Cytopathic effects (CPE) were monitored daily, and the number of CPE-positive wells was recorded. The 50% tissue culture infectious dose (TCID_50_) was calculated using the Reed-Muench method.

### Pull-down

Eukaryotically expressed and purified PDCoV S protein fused with a Strep tag II was treated separately with buffers at pH 7.2 and pH 4.0. After neutralization, the S protein was incubated with streptactin agarose resin 4FF at 4°C for 4 h to allow sufficient binding to the resin. Following incubation, unbound S protein was removed by washing with five column volumes of PBS. Eukaryotically expressed and purified His-tagged pAPN protein was then added, and the mixture was incubated at 4°C for 12 h. After incubation, unbound proteins were washed off with 10 column volumes of PBS. The protein complexes were subsequently eluted with D-biotin buffer and analyzed by SDS-PAGE.

### SPR assay

Protein binding affinity was measured using a Biacore 8K system at room temperature. Briefly, pAPN protein was immobilized on a CM5 sensor chip (GE Healthcare, USA) via amine coupling at an immobilization level of approximately 2,100–2,200 response units. Multi-cycle kinetic assays were performed with two analytes, S7.2 and S4.0. HEPES buffer (GenScript, China) was used as the running buffer, and serially diluted S7.2 and S4.0 proteins were sequentially injected over the chip surface, with binding response signals recorded in real time. All sensorgram data were subjected to kinetic fitting and analysis using Biacore 8K Evaluation software version 5.0.

### Indirect ELISA

Protein samples were diluted to 5 μg/mL in 50 mM carbonate buffer (pH 9.6) and added to 96-well microplates (Corning, USA) at 100 μL per well. Coating proceeded overnight at 4°C. After removal of the coating solution, the plates were washed three times with PBST and blotted dry on filter paper. The wells were then blocked with 5% BSA in PBST for 2 h. After removal of the blocking solution, plates were washed three times with PBST and blotted dry on filter paper. HRP-conjugated pAPN was serially diluted (1:100, 1:200, 1:400, 1:800, 1:1,600, 1:3,200, 1:6,400, and 1:12,800), and 100 μL of each dilution was added to the wells and incubated at 37°C for 1 h. Next, the plates were washed five times with PBST, and 100 μL TMB substrate solution was added to each well and incubated for 15 min at 37°C. The reaction was terminated by the addition of 100 μL of 2 M H₂SO₄ to each well, and absorbance was measured at 450 nm with a microplate reader.

### Piglet challenge experiment

Twenty-two 5-day-old suckling piglets were purchased from a commercial farm with no history of PDCoV infection or vaccination. Before challenge, serum and rectal swab samples were collected and tested to confirm the absence of PDCoV-specific antibodies and antigens. All piglets were randomly assigned to three groups: a treated group (*n* = 8), an untreated group (*n* = 8), and a mock control group (*n* = 6). The piglets were housed separately by group in individual pens and cages. One day before the challenge, piglets in the treated group were orally administered Al(OH)₃ gel three times (3 mL per dose per piglet), whereas those in the untreated group received PBS, and those in the mock control group received no treatment. After the three administrations, two piglets in the treated and untreated groups were randomly selected for euthanasia, and gastric fluid was collected to measure pH. Subsequently, piglets in the treated and untreated groups were orally inoculated with 1 mL of PDCoV CH/XJYN/2016 viral suspension (10^4.80^ TCID_50_/mL), whereas the mock control group remained unchallenged. At 18 h and 24 h post-infection, intestinal tissues were collected for RT-qPCR analysis and immunohistochemical analysis.

### Histopathology and immunohistochemistry

At 18 h post-challenge, segments of the small intestine were collected from piglets and fixed in 4% paraformaldehyde. Fixed tissues were processed according to standard histopathological procedures, including dehydration, trimming, paraffin embedding, sectioning, hematoxylin staining, mounting, and microscopic examination. A monoclonal antibody to the PDCoV N protein, previously prepared and preserved in our laboratory, served as the primary antibody, whereas HRP-conjugated goat anti-mouse IgG served as the secondary antibody. Images were acquired with a Pannoramic 250 digital slide scanner and a BA200Digital trinocular microscope imaging system.

### Negative-staining transmission electron microscopy and data processing

Protein samples were first assessed for purity with SDS-PAGE, then used for preparation of negatively stained grids. Copper grids coated with a continuous carbon film were glow-discharged (15 mA for 60 s) to render the surface hydrophilic. With fine self-locking straight tweezers, a 4 μL aliquot of protein sample was applied to the grid and incubated for 1 min. Excess liquid was removed from the edge of the grid with filter paper, thus leaving a thin film of sample. The grid was then floated on a 20 μL drop of 3% (w/v) uranyl acetate for 10 s, and this staining procedure was repeated three times. After a final incubation for 30 s, excess stain was blotted off with filter paper, and the grid was air-dried for 15–20 min. The prepared grids were observed with a Talos F200S transmission electron microscope operated at 200 kV. Images were acquired from regions exhibiting optimal staining and particle distribution at appropriate magnifications.

### Cryo-electron microscopy data collection, processing, and model building

Cryo-EM grids were glow-discharged at 15 mA for 40 s to render the surface hydrophilic. A 4 μL aliquot of protein sample was applied to the treated grids, which were then vitrified with a Vitrobot under conditions of 4°C and 100% humidity, rapidly plunged into liquid ethane, and stored in liquid nitrogen. Frozen samples were transferred under liquid nitrogen to the holder, mounted onto a C-ring, and loaded into the autoloader for data acquisition with a 300 kV cryo-electron microscope. Grid quality was systematically evaluated, including ice thickness, ice quality, particle distribution, sample concentration, and particle homogeneity. After selection of suitable grids, data collection was performed. Image processing was performed with RELION 4.0 or cryoSPARC. Model refinement was performed with Phenix, model building was conducted in Coot, and structural visualization and figure preparation were performed with UCSF Chimera or PyMOL.

### Statistical analysis

Statistical analyses were performed in GraphPad Prism 8.0. Data from three independent experiments were analyzed statistically. Statistical significance was determined with unpaired two-tailed *t*-tests. Data are presented as the mean ± standard deviation with 95% confidence intervals. Differences were considered statistically significant at *P* < 0.05. Significance levels are indicated as follows: *P < 0.05, **P < 0.01, and ***P < 0.001.

## Data Availability

All relevant data are within the paper and are available upon request from the corresponding authors.

## SUPPLEMENTAL MATERIAL

Supplemental material jvi.01168-26-s0001.docx Fig. S1 to S4.

ASM does not own the copyrights to Supplemental Material that may be linked to, or accessed through, an article. The authors have granted ASM a non-exclusive, world-wide license to publish the Supplemental Material files. Please contact the corresponding author directly for reuse.
