# Supplementary material for: Low-pH-induced conformational changes in the spike protein promote porcine deltacoronavirus infection by enhancing viral binding efficiency to the porcine aminopeptidase receptor
Source: J Virol. 2026 Sep 9;100(10):e01168-26. doi: 10.1128/jvi.01168-26 (PMC13712870; doi:10.1128/jvi.01168-26)
Supplement: Supplementary file 1 [file jvi.01168-26-s0001.docx]

**Low-pH-Induced Conformational Changes in the Spike Protein Promote Porcine Deltacoronavirus Infection by Enhancing Viral Binding Efficiency to the Porcine Aminopeptidase Receptor**

Ruiming Yu^1,2,3^, Liping Zhang^1,2^, Peng Zhou^1,2^, Zhongwang Zhang^1,2^, Yingjie Bai^1,2^, Dongsheng Wang^1,2,3^, Ya Liu^1,2^, Xinru Wang^1,2^, Gang Du^4^, Ligang Yuan^3^, Huichen Guo^1,2^, Li Pan^1,2^, Xinsheng Liu^1,2,^*

^1^ State Key Laboratory of Animal Disease Control and Prevention, College of Veterinary Medicine, Lanzhou University, Lanzhou Veterinary Research Institute, Chinese Academy of Agricultural Sciences, Lanzhou 730046, China..

^2^ Gansu Province Research Center for Basic Disciplines of Pathogen Biology, Lanzhou 730046, China.

^3^ College of Veterinary Medicine, Gansu Agricultural University, Lanzhou 730070, China.

^4^ Department of Ophthalmology, The First Hospital of Lanzhou University, Lanzhou 730000, Gansu, China.

Authors' email addresses:

Ruiming Yu: yuruiming1992@163.com

Liping Zhang: [zhangliping03@caas.cn](mailto:zhangliping03@caas.cn)

Peng Zhou: [zhoupeng02@caas.cn](mailto:zhoupeng02@caas.cn)

Zhongwang Zhang: [zhangzhongwang@caas.cn](mailto:zhangzhongwang@caas.cn)

Yingjie Bai: [baiyingjay@163.com](mailto:baiyingjay@163.com)

Dongsheng: Wang: [wangdongsheng0728@126.com](mailto:wangdongsheng0728@126.com)

Ya Liu: [liuya7793@163.com](mailto:liuya7793@163.com)

Xinru Wang: 15993521586@163.com

Gang Du: dugang705@163.com

Ligang Yuan: yuan2918@126.com

Huichen Guo: guohuichen@caas.cn

Li Pan: [panli@caas.cn](mailto:panli@caas.cn)

Xinsheng Liu* (corresponding author): liuxinsheng@caas.cn

**Figure S1 SDS-PAGE analysis of the PDCoV S trimer.** (A) The purified PDCoV S trimer was subjected to SDS-PAGE and stained with Coomassie brilliant blue. (B) Negative-stain transmission electron microscopy validation of low-pH-treated PDCoV S trimers. Left, a representative overview micrograph showing uniformly dispersed, intact spike particles with no obvious aggregation. Scale bar, 100 nm. Right, representative two-dimensional (2D) class averages reveal well-preserved prefusion trimer architecture with consistent conical morphology across all particle classes.

**Figure S2 Cryo-EM data analysis of PDCoV S4.0.** After 300 kV cryo-EM data collection for PDCoV S protein (pH 4.0), motion correction, and CTF estimation, 3,032,924 particles were selected for 2D classification. After several rounds of 2D classification, 993,371 particles were classified in 3D, and the S protein was in a pre-fusion state. Finally, 251,057 particles were selected for homogeneous refinement and non-uniform refinement, and the Refine map was finally obtained. After Sharpen was applied, the map was used to build the final PDCoV S protein model.

**Figure S3 PDCoV S protein structure S4.0 at 2.83 Å resolution.** Gold-standard Fourier shell correlation (FSC) curves of S4.0. Local resolution map of S4.0 density map analyzed with the local resolution estimation tool in cryoSPARC.

**Figure S4 Overall structure of the PDCoV S protein trimer at low-pH (pH 4.0).** (A) Schematic diagram of PDCoV S protein organization. Different domains are displayed in different colors. (B) Orthogonal view of the three-dimensional structure of S protein. Protomers A, B, and C are cyan, magenta, and green, respectively. (C) The atomic structures of the three-dimensional structure of S protein are shown in cartoon representation. The domains are indicated and colored as defined in (A).
